# Practical Observations on Various Subjects Relating to Midwifery

**Published:** 1837-01

**Authors:** 


### Art. VII.

*Practical Observations on various Subjects relating to Midwifery.* By James Hamilton, m.d. f.r.s.e., Professor of Medicine and Midwifery, &c. in the University of Edinburgh. Part I.—*Edinburgh*, 1836. 8vo. pp. 317.

It is not necessary for us to make any formal efforts to attract the attention of our readers to a work coming from the pen of so respected and able a practitioner and so well-known a teacher as Dr. Hamilton, of Edinburgh. From him many of the most celebrated accoucheurs of the present day, in Great Britain and Ireland, derived their first obstetrical instruction; and every one who was his pupil must readily admit that no man can be more dexterous in the difficult task of conveying solid and good practical instruction in an amusing and attractive manner. The object of the volume before us is to record these deviations from the established modes of practice, in several ordinary affections of women, which the experience of nearly half a century has led the author to adopt and recommend. By pursuing such a plan, a veteran practitioner like

Dr. Hamilton promises to be much more useful to his juniors, and to the profession at large, than if he were to undertake a general treatise upon the subject to which he has devoted his attention; for then his original views would be lost, or at least obscured in the mass of matter which he must necessarily compile from various writers. The subjects discussed by the author are few in number, and therefore we shall at once enumerate them. The volume comprises chapters on the Prolapsus of the Uterus; Polypous Excrescence of the Uterus; Enlargement of the Ovary; Evidences or Signs of Human Pregnancy; Duration of Human Pregnancy; the Management of the first, second, and third Stages of Labour. In an Appendix, too, is given a report of experiments, with the Stethoscope, on the Action of the Fœtal Heart, by Dr. JOHN MOIR. In giving an abstract of Dr. Hamilton's well-matured opinions upon these different topics, we shall take care that whatever critical comments we may introduce, shall be tempered by that deference which he may fairly claim, while they will be free from that subserviency which he would rather deprecate than approve, and which we are not inclined to show even to the doctrines of a master.

*Prolapsus of the Uterus.* Dr. Hamilton expresses his astonishment that medical practitioners should have fallen into the most extraordinary errors respecting both the nature and treatment of this ailment, considering the frequency with which it presents itself to their notice. The incipient symptoms of prolapsus uteri are well known; that they vary in different individuals is as well understood. But the irregular progress of the disease in different individuals is not always impressed upon the mind of the practitioner. In robust women of the lower ranks, little inconvenience is often experienced till the uterus is actually protruded through the external parts; and even then, by any mechanical contrivance to prevent the actual protrusion of the organ, they are capable of fulfilling laborious duties. In delicate females of the higher ranks, the uneasy feelings on standing or walking lead them to avoid all exertions. Their health declines for want of air and exercise, and the increasing descent of the uterus produces an unusual discharge from the mucous glands of the vagina. Thus the general weakness is aggravated, and a broken constitution follows. Dr. Hamilton doubts the accuracy of the general opinion, that the sufferings of the patient are proportioned to the degree of the disease. His experience teaches him that peculiarity of constitution has more influence than the degree of displacement, and he corroborates this opinion by the brief mention of different cases.

The diagnosis of the disease is easy, if the proper measures are taken. "There are at least three diseases with which prolapsus uteri may be confounded, and from which, of course, it is necessary to distinguish it, viz. chronic enlargement of the uterus, polypous excrescence, and incipient scirrhosity. Nothing but actual examination can enable the practitioner to draw the line of distinction." (P. 6.) The prognosis may be always favorable as far as life is concerned. The probability of a radical cure depends on the age of the patient, her general health, the apparent cause of her disease, and its duration. In elderly relaxed women relief may be afforded, but nothing more should be expected or promised. In young healthy subjects, a complete cure may be expected, unless from any cause some of the parts which naturally support the womb are destroyed.

It was once the universal, and is now the very general opinion,* that prolapsus of the uterus was principally owing to relaxation of the ligaments that support it. Dr. Hamilton believes, with Gardien,† that the ligaments of the uterus do not retain it in its natural situation. It will be found, he argues, that in any case of prolapsus uteri, the vagina or bladder, or rectum, or muscles lining the pelvis, or filling up its outlet, are debilitated or lacerated, and therefore the relaxation of the peritonæum and its productions (the ligaments of the uterus) is the effect of the prolapsus, and not the cause. The refutation of some apparent objections to this doctrine is not omitted. Dr. Hamilton will be gratified to know that he is supported in these views by one of the first authorities in Germany, Osiander.‡

The treatment hitherto pursued in cases of prolapsus uteri has been the following: viz. in incipient cases, the horizontal posture, the application of cold to the loins, external parts, and vagina; the injection of styptic liquors into the vagina; tonic medicines; and, in cases of long standing, in addition to the above means, pessaries. Dr. H. admits that the horizontal posture immediately relieves the uneasy feelings of the patient, but he long ago ascertained that it tends not only to impair the general health, but also to aggravate the disease, by increasing the relaxation of the natural supports of the womb; and "daily experience has established the validity of this opinion." It must be evident that the *constant* observance of the horizontal position (against which, we presume, the author contends,) will be more or less injurious to the general health; but such evil effects would not arise from the patient lying down for some hours a day, while the cure of the prolapsus would be greatly facilitated, as far as we can judge from the great difficulty of affording relief in cases where the situation and occupation of the patient prevent her from remaining in the horizontal position for a great part of her time.

"The application of cold, either in the form of the cold bath, or by lotions of water artificially cooled, have been highly extolled by Sir Chas. M. Clarke. In slight cases the cold plunge bath furnishes an excellent auxiliary means; but the author cannot sanction the introduction of a piece of ice into the vagina, as suggested by Sir Charles. It could answer no good purpose, that is, it could not cure the disease, while the probability is, that it would produce inflammation of the surface of the vagina. As to the injecting of styptic liquors into the vagina, it is a practice to which also the author, from much experience, must object in the most explicit terms." . . . . "Against this mode of practice the author has to offer the following, as he considers, most serious objections:—1. On the supposition that styptic injections were safe, and that they could really restore tone to the vagina (which the author concedes for the sake of argument, for the contrary is his sincere belief,) it must be obvious, that if his view of the nature of the disease be correct, no benefit could accrue from the practice. Accordingly, no practitioner trusts to those means in cases of any considerable degree of prolapsus uteri. 2. It is admitted that, as the irritability of the mucous membrane of the vagina varies in different women, as well as in the same woman at different periods of time, the injection of strong astringents may prove injurious. Doubts are, therefore, entertained on the safety of the practice, even by those who recommend it. 3. The author's experience has convinced him, that astringent injections into the vagina

* Obs. on the Dis. of Females, by Sir C. M. Clarke, Part I., p. 68. Burns' Midwifery, 8th Edit. p. 130. Dr. D. Davis's Principles of Obstetric Medicine, p. 548, &c.

† Accouchemens, t. i. p. 177.

‡ Die Ursachen und Hülfsanzeigen der unregelmässigen und schweren Geburten, von Dr. J. Osiander. Zweite Auflage. Tübingen, 1833. B. 3, s. 130.

are apt to injure the uterus, rather than the canal into which they are thrown. He can solemnly aver, that of the numerous cases of chronic enlargement of the uterus which have fallen under his notice, by far the greater number had been unequivocally occasioned by the use of styptic injections per vaginam. 4. The immediate effect of such injections in cases of prolapsus uteri of any standing, viz., the diminution or suppression of leucorrhœal discharge, has been in many cases followed by distressing headachs, or obstinate inflammation of the eyes, or eruptions on the face." (P. 15.)

We cannot say that we have seen any very decided advantages from the use of styptic injections in cases of prolapsus; for their employment is seldom trusted to alone; neither did we ever see the injurious effects ascribed to them by Dr. H. Internal tonics are very useful auxiliaries.

We are by no means convinced by the arguments so strongly urged by Dr. Hamilton against the employment of pessaries in cases of prolapsus. They appear rather directed against the abuse than the judicious use of these instruments, from which, in many cases, we have found such decided advantage, and without "subjecting the patient to the charge of the medical attendant for life," or even a long period: so that we really cannot consent to give them up ourselves, or to recommend others to do so, even upon the authority of our author. We do not feel ourselves called upon to enter fully into the subject of the employment of pessaries in prolapsus uteri. Most elementary works on the Diseases of Women lay down the mode of applying them, and the cases in which they are proper. In justice to Dr. Hamilton, we must observe that he is again supported by Osiander;* but in our opinion the German as well as the English professor has been much too hasty and too exclusive in his condemnation of an instrument which, when skilfully employed, is certainly not liable to produce the mischievous effects which they attribute to it. As Dr. H. feels convinced that the established practice in cases of prolapsus uteri is most unsuccessful, he has suggested other means of cure. Observation and reflection led him to the discovery of a mode of supporting the uterus which is both effectual and safe, "and the experience of several years has now fully established its superiority to every means hitherto suggested." It consists in the use of the T bandage, with a cushion interposed between the outlet of the pelvis and the cross strap of the bandage. "In every case of prolapsus, whatever may have been its degree, to which he has been called for several years, he has suggested this very simple contrivance." Different modifications of the bandage are described by the author which are required in different cases of the disease. Osiander,† as firm an opponent to common pessaries as Dr. Hamilton, recommends, as "more appropriate, less dangerous, and giving hopes of a perfect cure even in very old cases," the following plan: Two fine linen bags, about two fingers broad, and four or five inches long, are to be filled with very finely powdered oak-bark. Before they are used, they are to be steeped some hours in red wine. One of these bags is to be introduced into the vagina in the morning, and the other in the evening; and, to prevent their falling out, a T bandage is to be applied over the genital organs. Osiander assures us that, by the regular continuance of this plan from two to three weeks, *the patient remaining as much as pos-*

* Loc. cit. p. 134.

† Loc. cit. 135.—Siebold never uses hard pessaries. Journ. für Geburtshülfe, &c. 1822. B. 3, s. 40.

*sible in a recumbent position,* the relaxed and expanded vagina will be so much constricted, that, even in cases where the whole hand could be before introduced without any difficulty, there will be hardly space for two fingers, and that thus the disposition to prolapsus will be removed.

"Walking exercise," according to Dr. Hamilton's experience, "is the most powerful means which can be suggested for strengthening the natural supports of the uterus. Of course where the patient has been much debilitated, or has been long confined to the horizontal posture, this exercise must be cautiously begun; but, whatever be the feelings of the patient, it must be gradually increased till it equal that which an individual usually takes in the ordinary state of health." (P. 30.)

As far as we know, this mode of managing a patient with prolapsus uteri is quite original. In very many cases the patient neither could nor would submit to it, and we strongly doubt whether it can in any case be proper. It cannot be doubted that the total want of exercise, to which many persons with this disease are subjected, is a great evil, and most prejudicial to the general health, and consequently to a perfect cure; but we cannot, even on Dr. Hamilton's authority, feel justified in recommending his mode of treatment without some personal experience of its safety. We however recommend the subject to the earnest consideration of our readers, and shall certainly on a fit occasion give it a fair trial. Cold bathing and internal tonics are approved of as auxiliaries.

*Polypous Excrescence of the Uterus.* Dr. Hamilton confirms the opinion of Dr. Gooch and other experienced writers, that many women die of this disease, in whom the nature of the complaint has not been even suspected; for in many cases the symptoms are, if not obscure, at least easily mistaken for other maladies. The most ordinary symptom of polypus of the uterus is an increased flow at the usual menstrual periods, accompanied sooner or later with a discharge of coagula. Leucorrhœa, pain in the back, and a sense of pressure or bearing down, supervene after some time in most cases.

"In the further progress of the disease, the draining from the vagina increases in quantity, and becomes acrimonious and offensive. When this change happens, the state of the general health is rapidly impaired, œdematous swellings of the lower extremities follow, and if no effectual means be employed, the patient sinks exhausted." (P. 36.)

Sometimes many months elapse after the frequent uterine hemorrhage had indicated the existence of the disease, before any draining from the vagina, during the intervals between the menstrual periods, takes place.

In proof of this, the author mentions two cases in which the polypus was as large as "a new-born infant's head." (P. 38.) It might be supposed that, when the polypus has attained a certain size, it must occasion pressure or bearing down, and yet neither symptom invariably happens. This we know from our own observation in public and private practice.

"The patient from whom the largest polypus in the author's possession was taken, had, within a few weeks before falling under his care, walked up and down some of the highest mountains of Britain without inconvenience or fatigue." (P. 39.)

Dr. Gooch agrees with Dr. Baillie, that the "internal structure of polypus in most cases exactly resembles the internal structure of the large white tubercle of the uterus, commonly called the fleshy tubercle. "They are, (he says,) the same disease, differing only in the seat and mode of their attachment, and consequently in the symptoms which they produce." Such is certainly the structure of the majority of polypi we ourselves have seen, but Dr. Hamilton regards such characters of the excrescence as exceptions to a general rule. "Polypous excrescences when of a large size are commonly of a soft fibrous texture, with numerous loaded veins on their surface." Dr. Hamilton's experience leads him to believe that hemorrhage from the surface of the polypus is rare.

"In no instance to which he has been called has there ever been any bloody discharge from the surface of the polypus, notwithstanding any liberty he might have taken in pressing upon it, or in attempting to twirl it round." (P. 43.)

Dr. Gooch* is opposed to this opinion. Burns† partly coincides with it. We, with Dr. Hamilton, do not remember to have seen blood discharged from the surface of the polypus, however roughly it was handled. "The locality of these excrescences has perhaps more influence upon the symptoms of the disease than either the size or texture." Polypus can only be distinguished by actual examination; and, even with this assistance, the young practitioner may be mistaken in his diagnosis. In proof of this we give the following case from M. Velpeau, and cannot refrain from remarking that the candour with which it is related is highly creditable to him. A woman, aged forty, presented herself at a Paris Hospital. She said she had prolapsus uteri, for which she had worn a pessary for two years, but which she had neglected for fifteen months, as the instrument was mislaid. The tumour was found, upon examination, to protrude from the vagina about two inches: it was easily returned, was of a conical shape, and had a transverse cleft at its most depending part, so as to present two unequal lips, the anterior one of which was a little longer than the posterior. The neck of the tumour was narrowed a little above the vulva. The mistaken diagnosis of the case was strengthened by the declaration of the patient that she menstruated from this orifice or cleft in the tumour. M. Bourgon desired M. Velpeau to apply a pessary. Soon after the patient died of peritonitis, and, upon dissection, it was ascertained that the imagined prolapsus uteri *was a polypus* attached to the fundus of the cavity of the uterus. The preparation is in M. Velpeau's collection, and it is very remarkable from the perfect similarity that exists, at the inferior part of the tumour, to the os uteri. Upon the surface of polypous tumours, depressions or furrows occasionally exist, which, although less deceptive than the appearance in M. Velpeau's case, might easily deceive an inexperienced practitioner.‡

Dr. Hamilton enters at some length on the subject of diagnosis, which, we may remark, is still more fully discussed by Gooch§ and Madame Boivin.||

Respecting the prognosis in cases of polypus uteri, the author's experience leads him to differ from Dr. Gooch, who says, "if mistaken and neglected, it occasions the death of the patient; if detected and removed, she not only lives but regains perfect health." To this statement Dr. H. objects, that it is too strongly expressed; for, conceding that neglected

* On some of the most important Diseases of Women, p. 260.
† Midwifery, 8th Edit. p. 115.
‡ Anatomie Chirurgicale, 2d Edit. t. 2, p. 362.
§ Loc. cit. 299.
|| On Diseases of the Uterus. Translated by Heming, p. 201.

cases usually end fatally, yet sometimes, by an effort of nature, the polypus is separated and expelled, either in the act of vomiting, or by strong expulsive uterine pains.

"In the author's collection, there is a very large polypus, which had been thus naturally thrown off in the case of an unmarried lady; and her health, which had been previously much impaired, was completely restored." (P. 59.)

On the other hand, the removal of the tumour, even though safely effected, does not invariably secure the recovery of the patient. In support of this opinion three cases are related. But we must observe, that in all these cases the ligature was employed; in the two first it is stated that the patients died of enteritis; and it is probable that the same result caused the fatal termination of the third. Now, if the polypi had been removed by excision, each of the patients would, in all probability, have been saved. Again, Dr. Gooch thought so little of the difficulty of applying the ligature, that he states "any surgeon with a proper instrument is competent to remove the polypus." Dr. Hamilton, on the contrary, and we are sure correctly, says, that admitting the general success of the operation, when properly performed, it is in many cases one of the most difficult and dangerous operations of surgery. He has not only "operated on several patients who had been dismissed from public hospitals as incurable, but he has seen some of the most eminent practical surgeons of this part of the kingdom foiled in their endeavours to apply the ligature." (P. 64.) We must be permitted to point out two errors contained in the following statement.

"British practitioners have now universally agreed that the safe mode of operating in those cases is by ligature, though several eminent French surgeons have lately preferred the double operation of tying the polypus, and then cutting it off." (P. 65.)

So far from British surgeons having "universally agreed" to the use of the ligature, excision by the knife or curved scissors, the latter being decidedly the safest and most convenient instrument, is now very generally preferred. Again: the French surgeons of the present day follow the example of Dupuytren, who successfully and safely removed upwards of two hundred uterine polypi by excision, *without* any application of the ligature. The "double operation" is very rarely had recourse to by them. The operation of excision,* which was recommended by the ancients, alarmed practitioners in consequence of the fatal hemorrhage which ensued in the case recorded by Zacutus; but this is a solitary case, notwithstanding the numerous instances in which it has of late years been performed. M. Dupuytren was the first to return to the former practice of excision; it is also adopted by Osiander, Siebold, Mayer, and indeed the great majority of modern surgeons. Mr. Arnott recently made the removal of uterine polypi the subject of a clinical lecture at the Middlesex Hospital, which he has since published.† In this lecture, in which he details his own experience, will be found a very clear and practical statement of the *general* superiority and greater safety of excision than of the ligature. And, as we have already hinted, the fatal result of the cases related by Dr. Hamilton himself, at page 59 et seq., goes very far to show that, *generally*, excision is to be preferred to the ligature.

* Boivin and Dugès, loc. cit. p. 211.

† Medical Gazette, June 11, 1836, p. 410.

We believe that if, in these instances, the polypi had been dexterously removed by excision instead of the ligature, no attack of enteritis would have followed.

*Enlargement of the Ovary* is the next subject considered by the author. The chapter is interesting and full of practical information. In most cases the disease has made great progress before either the patient or the practitioner is aware of its existence. Few local diseases vary so much in their progress in different cases. Dr. Hamilton states, and we have seen several cases which bear him out in the assertion, that not unfrequently the mere enlargement of the ovary neither injures the health of the patient nor appears to shorten her life. On the other hand, in many instances, after a certain progress, painful and alarming symptoms suddenly supervene, and prove rapidly fatal. A very interesting case recently occurred in our own practice, which shows not only the obscure character of the incipient symptoms of even malignant enlargement of the ovaria, but also the suddenness with which the disease sometimes takes an alarming turn, and the rapidity with which it destroys the patient, when no danger had been apprehended. A young lady, about twenty-eight years of age, who had always been delicate in appearance, but who still enjoyed good health, was attacked, without any obvious cause, with pain in the back, some pain in the thigh, and occasional vomiting. These symptoms, the only ones complained of for several weeks, continued in spite of different remedies prescribed by another practitioner. When the patient was placed under our care, her countenance certainly bore the well-known traits of organic mischief, and she looked like a person affected with uterine disease: the complexion was of a sallow, leaden hue, and the features expressive of great depression of strength. Her spirits were, however, very cheerful, and neither her friends nor herself apprehended the existence of any formidable disease. We were nevertheless led, by the circumstances just mentioned, to enquire if she had ever felt pain in the lower part of the abdomen, or if any symptoms had existed of uterine or ovarial disease. Neither the one nor the other had occurred. Upon making an examination, however, we found a very considerable enlargement of the right ovary, which was somewhat painful on pressure; and in various parts of the abdomen hard tumours could be felt, some of which were slightly painful. It is worthy of attention, that the patient was quite unconscious of the existence of the tumours in the abdomen and ovarial region. With this evidence, we gave it as our opinion that the enlargements were of a malignant character, and that sooner or later the case would terminate fatally. In the course of a few days the tumour of the right ovary became very painful, and great pain and distress were complained of in the abdomen generally. The treatment adopted was of little or no avail, and in about three weeks the patient died. Upon dissection, we found both ovaria greatly enlarged, the disease being evidently that commonly termed Fungus hæmatodes.

Dr. Hamilton mentions two cases in which a spontaneous disappearance of enlargement of the ovaria took place. Two examples of this kind have also occurred in our own practice, in each of which there was decided enlargement of one ovarium, which gradually disappeared, without having apparently been influenced by medical treatment, or causing much derangement of health.

"Several individuals, within the author's knowledge, have dragged on a miserable existence under this disease for between twenty and thirty years, although the bulk had become so great that the size of the belly equalled that of a pregnant woman at the full time. These discrepancies in the progress and symptoms of the disease are explained only by what is observed after death; for it seldom happens that, during life, there are any marks by which the probable course of the disease can be foretold." (P. 75.)

Several cases are mentioned by the author, which prove the difficulty that not unfrequently exists in distinguishing ovarial disease from other disease, and vice versâ.

The prognosis in cases of diseased ovary is more difficultly formed than in almost any other organic disease. "Indeed, it is impossible, in any given case, to foretel the probable result." This is expressing the opinion perhaps rather too strongly; for we may sometimes determine that the case will rapidly kill the patient, and sometimes that it will not in all probability destroy her for years. After referring to the general opinion that we can hope to do no more than palliate symptoms in cases of ovarial disease, Dr. H. states that "he can prove, by many living witnesses, that cases now and then occur where the disease is curable, not merely in its early stage, but after it has attained such a magnitude as to require the operation of tapping." (P. 97.) About twenty years ago he was induced by particular circumstances to make some experiments, for the purpose of determining whether ovarial enlargements could possibly be removed; and, in doing so, he paid every attention to the general health of the patient.

"Adverting to the effects of percussion and of pressure in chronic rheumatism, and knowing the influence of the continued use of the muriate of lime in indolent glandular swellings, he was led to the trial of those several means, as being at any rate perfectly safe. He advised, therefore, that moderate and equable pressure of the abdomen should be made by means of a suitable bandage; that the enlarged part should be subjected twice a day to gentle percussion, and that a course of small doses of muriate of lime should be continued for at least several months. Where pain or tenderness was experienced on the ovary being pressed upon, he recommended, in addition to the above means, the daily use of the warm bath." (P. 99.)

This plan of treatment, Dr. H. assures us, has been much more successful than he anticipated. In seven cases in which it has been tried, the enlargement completely subsided. In the majority of these cases there could be no mistake, as the size of the ovary was considerable, and the fluctuation distinct. Previous to the diminution of bulk in all the successful cases, the enlargement of the ovary became soft. The practice thus adverted to was mentioned by the author in a work* published many years ago, and since that time his additional experience has confirmed his confidence in it. A somewhat peculiar mode of treating the disease, pursued by Dr. Barlow, of Bath, is noticed in another part of the present Number, p. 165. Dr. Hamilton considers the operation of tapping unsafe till the ovarial sac has acquired a certain degree of distention. He urges various, and to us satisfactory, objections to the operation of extirpating enlargements of the ovaria, and deprecates the practice proposed by Sheldon, of keeping up a constant discharge by

* Observations on the Use and Abuse of Mercurial Medicines in various Diseases, p. 200.

means of blistering over the enlargement, and dressing with savine ointment. He is also opposed to another plan which has been adopted,—that of passing a seton through the tumour; and places no confidence in Dr. Jenner's suggestion of keeping up a constant state of nausea for a considerable time.

*Evidences or Signs of Human Pregnancy.* The author's observations are brief upon this often debated and still debateable subject. In our opinion, he assumes in the following sentences more than the present state of science will justify, or than the experience of most practical men will support. "He undertakes to prove that, both in the early and in the latter months of pregnancy, there are invariable signs marking that condition of the system."—"The author has no hesitation in asserting that there are two circumstances which invariably attend pregnancy during the early months; viz. suppression of the catamenia, and a perceptible change on the surface of the mammæ surrounding the nipple." He admits that all other symptoms are liable to so much variation in different individuals, and even in the same individual in different pregnancies, that they ought to be disregarded. The "*invariable*" suppression of the catamenia during the early months of pregnancy is positively denied by the very great majority of modern writers; and scarcely any experienced practitioner with whom we have conversed upon the subject has not known, as we certainly have ourselves in several well-marked cases, menstruation continue regularly, but generally in diminished quantity, during the period referred to. Without unnecessarily accumulating evidence upon this very important subject, which is readily accessible to every enquirer, we content ourselves with referring to Dewees,* Blundell,† Velpeau,‡ and Montgomery.§ The very general suppression of the menses during pregnancy is universally admitted; but in the present day there are very few who would advocate, with Dr. Hamilton, the "*invariable*" existence of this. So also with respect to the discoloration and altered appearance round the nipple. We admit that the peculiar appearance of the areola pointed out by Dr. Hamilton, especially when combined with suppression of the catamenia, affords strong presumptive evidence of the existence of pregnancy; but we must object to its being laid down as an unerring proof of pregnancy. In this opinion also, which our own experience convinces us is well founded, we are supported by almost all practical writers and observers.

"As to the areola being formed in many of the complaints which resemble pregnancy, as Dr. Denman at one time alleged, it is unnecessary to make any other remark than that it is quite inconsistent with the observation of every modern practitioner." (P. 143.)

Dr. Denman's opinion is not at all inconsistent with our own observation. We have seen, in cases of dysmenorrhœa especially, those changes of colour and appearance around the nipple which might easily have been mistaken for signs of pregnancy; and we know that other practitioners of experience have seen the same changes occur from dysmenorrhœa, and even simple uterine irritation. Our author likewise

* Midwifery, first edition, p. 93. † Principles of Obstetricy, p. 164.
‡ Traité des Accouchemens, t. i. p. 182.
§ Cyclopædia of Pract. Med., art. "Signs of Pregnancy," vol. iii. p. 470.

believes, almost alone, that in the latter months "the movements of the infant can *always* be distinguished by an attentive practitioner." We sincerely wish we could assent to his opinion upon these "invariable" signs of pregnancy, either in the early or latter months; but we cannot go further than to admit them as very valuable and trust-worthy guides *in general.* As Dr. H. "has not met with a case during the last thirty years where he could not ascertain pregnancy after the fifth month, when the infant continued to live, by the marks suggested in the preceding observations," he has had no opportunity of verifying the allegations of Kergaradec and others, who have practised auscultation to solve the question of pregnancy.

In reference to the popular and even professional belief, that diseases occurring during pregnancy ought not to be treated in the usual manner, Dr. H. observes, that this opinion has always appeared to him erroneous, and he has seen many deplorable instances of its fatal consequences.

> "In acute diseases, the circumstance of the patient being pregnant ought to increase the activity of the practice, and in chronic diseases palliative means cannot be injurious. In chronic affections of the liver, the author would not put the patient upon a course of mercury if pregnant, but he would suggest means, for the relief of the complaint at least, which could be adopted with safety." (P. 164.)

The frequently contested question "of the duration of human pregnancy" occupies twenty pages of the work. Dr. H. admits that pregnancy in the human subject is occasionally protracted beyond the ordinary period, and he mentions cases in point; but he does not think himself entitled to give a decided opinion as to how long the protraction may be extended.

The last three sections of the volume contain Dr. Hamilton's opinions on the management of the first, second, and third stages of labour. The student and young practitioner will here find many hints that are worthy of their attention. It is well known that the mode of extracting the placenta in cases of morbid adhesion, which is usually adopted by British and foreign practitioners, is to insinuate the fingers between the substance of the after-birth and the surface of the uterus. Dr. Hamilton suggests a different, and, if it be effective, a certainly preferable proceeding. "When the symptoms denoting adhesion unequivocally occur, the practitioner must proceed instantly to relieve the patient. For this purpose the navel-string is to be held by the left hand, while the right hand is to be carried up, guided by it to the placenta. Pressure is now to be made upon its substance, bringing its circumference towards its centre, and detaching leisurely and carefully all that can be separated by this manipulation. The separated mass is now to be extracted by pulling by the navel-string with the left hand, while the complete contraction of the uterus is to be secured by suitable pressure with the right hand, which ought not to be withdrawn from the cavity till its parietes are in close contact." (P. 289.) In some cases even a large portion of the placenta may adhere so firmly to the uterus as not to admit of separation without much force, and consequently much danger. Many practitioners are so fearful of allowing even a small portion of the firmly adherent placenta to remain attached to the uterus, that they would, upon the principle of choosing the lesser of two evils, detach it with all the risk that the employment of manual force must incur. Dr. Hamilton "has attended many cases where two, three, or more days have intervened between the birth of the infant and the separation of the adherent indurated portion of the secundines, and he never witnessed any untoward symptom, such as flooding, or subsequent irritative fever. In one case, a mass weighing eight ounces was retained five days, without occasioning any symptoms indicating danger." (P. 298.) This exactly comports with our own experience, and, if our evidence were necessary in support of Dr. Hamilton's statement, we could relate several cases where we have deemed it more prudent to leave a portion of the placenta attached to the uterus, than to have recourse to what we consider the much more dangerous practice of removing it by manual force. In the course of the last few years many cases have been related, in which it has been said that even large portions of retained placenta were removed by absorption.* In one case which very recently occurred in our own practice, we have every reason to believe that this happened. The placenta was firmly adherent to the uterus, and we could not separate more than two-thirds of it with safety. We watched the case very carefully from day to day; the patient had no after-pains; there was no hemorrhage, nor were there the slightest appearances, upon the napkins, of portions of placenta which were subsequently expelled. In a fortnight the lady was quite well.

The preceding pages will sufficiently evince that the work of Dr. Hamilton contains much valuable matter; and, although the author certainly shows himself, on many occasions, much behind the present state of our knowledge, both in regard to the scientific and practical part of the departments of medicine on which he writes, we can still recommend his book as well deserving the notice of practitioners, both young and old.

---

### Art. VIII.

1. *St. Thomas's Hospital Reports.* No. III. and IV. *April and June,* 1836.

* Velpeau (loc. cit. t. ii. p. 534,) gives the best account of the published evidence upon the subject.

